# A Human *In Vitro* Whole Blood Assay to Predict the Systemic Cytokine Response to Therapeutic Oligonucleotides Including siRNA

**DOI:** 10.1371/journal.pone.0071057

**Published:** 2013-08-05

**Authors:** Christoph Coch, Christian Lück, Anna Schwickart, Bastian Putschli, Marcel Renn, Tobias Höller, Winfried Barchet, Gunther Hartmann, Martin Schlee

**Affiliations:** 1 Institute of Clinical Chemistry and Clinical Pharmacology, University Hospital Bonn, University of Bonn, Bonn, Germany; 2 Institute of Medical Biometry, Informatics and Epidemiology, University Hospital Bonn, University of Bonn, Bonn, Germany; McMaster University, Canada

## Abstract

Therapeutic oligonucleotides including siRNA and immunostimulatory ligands of Toll-like receptors (TLR) or RIG-I like helicases (RLH) are a promising novel class of drugs. They are in clinical development for a broad spectrum of applications, e.g. as adjuvants in vaccines and for the immunotherapy of cancer. Species-specific immune activation leading to cytokine release is characteristic for therapeutic oligonucleotides either as an unwanted side effect or intended pharmacology. Reliable *in vitro* tests designed for therapeutic oligonucleotides are therefore urgently needed in order to predict clinical efficacy and to prevent unexpected harmful effects in clinical development. To serve this purpose, we here established a human whole blood assay (WBA) that is fast and easy to perform. Its response to synthetic TLR ligands (R848: TLR7/8, LPS: TLR4) was on a comparable threshold to the more time consuming peripheral blood mononuclear cell (PBMC) based assay. By contrast, the type I IFN profile provoked by intravenous CpG-DNA (TLR9 ligand) in humans *in vivo* was more precisely replicated in the WBA than in stimulated PBMC. Since Heparin and EDTA, but not Hirudin, displaced oligonucleotides from their delivery agent, only Hirudin qualified as the anticoagulant to be used in the WBA. The Hirudin WBA exhibited a similar capacity as the PBMC assay to distinguish between TLR7-activating and modified non-stimulatory siRNA sequences. RNA-based immunoactivating TLR7/8- and RIG-I-ligands induced substantial amounts of IFN-α in the Hirudin-WBA dependent on delivery agent used. In conclusion, we present a human Hirudin WBA to determine therapeutic oligonucleotide-induced cytokine release during preclinical development that can readily be performed and offers a close reflection of human cytokine response *in vivo*.

## Introduction

Therapeutic oligonucleotides are a new promising class of pharmaceuticals with high potential in a variety of applications. Prominent examples are small interfering RNA (siRNA) used to inhibit the expression of a pathology driving gene [1] and immunostimulatory DNA/RNA (isDNA/RNA) that activate pattern recognition receptors (PRR) of the innate immune system as vaccine adjuvants or in order to treat infectious diseases and cancer [2], [3]. Known PRR that are activated by isDNA/RNA are the endosomally located Toll-like receptors (TLR) 3, TLR7/8 [4] and TLR9 (ligand CpG-DNA [2], [5], [6]), as well as the cytosolic helicases ‘Retinoic acid inducible gene-I’ (RIG-I) [7], [8] and ‘Melanoma differentiation associated gene 5’ (MDA-5) [9], [10] (respective oligonuceotide ligands see table 1). Whereas immune activation is the intended pharmacological mode of action for isDNA/RNA [3], it is an unwanted toxicological effect of siRNA, e.g. via TLR7 activation [4], [11], [12]. Systemic immune activation can lead to the severe, possibly life threatening condition of excessive cytokine release (cytokine storm) if not well understood and carefully controlled, as has been tragically shown in the past [13]. Since expression, ligand preference and function of the PRR is highly species-specific [3], cytokine release in humans is hard to predict in animal studies. Therefore, according to guidelines implemented after the Tegenero disaster (EMEA/CHMP/SWP/28367/07), novel therapeutic oligonucleotides possess characteristics of high-risk drugs when entering clinical development. Therefore, in addition to *in vivo* animal models there is a need for material saving, reliable, fast and easy to do *in vitro* tests in the human system during preclinical testing that predicts cytokine release in humans enabling a prediction of efficacy as well as a safe estimation of the first dose and the escalating dose steps for the phase I studies. So far, a whole blood assay (WBA) has been described in drug development as an easy-to-do *in vitro* test that closely mimics the human situation [14]–[17]. It is faster and cheaper than the more established peripheral blood mononuclear cell (PBMC) based assay and saves material since no isolation of cells is required. Immune activation by different drugs, including TLR ligands, has been tested [18]–[23] but it has not been evaluated for the special requirements in testing cytokine release induced by oligonucleotides, including oligonucleotides administrated with a delivery agent. Mostly Heparin, EDTA or citrate have been used as anticoagulants in WBA [18], [20], [23], [24]. Since anticoagulants can influence the immune system in several ways [25]–[27] it is unclear whether a WBA based on these anticoagulants reflects a physiological immune reaction. Here we aimed to establish a WBA specially designed for preclinical testing of cytokine release induced by therapeutic oligonucleotides.

**Table 1:** Overview of pattern recognition receptors and corresponding ligands used in this project.

pattern recognition receptors	ligand	
	oligonucleotide/nucleic acid	other
toll like receptor (TLR) 4	-	lipopolysaccharide
toll like receptor (TLR) 7	9.2s-RNA	resiquimod (R848)
toll like receptor (TLR) 8	9.2s-RNA	resiquimod (R848)
toll like receptor (TLR) 9	CpG DNA (CpG 2006; CpG M362)	-
retinoic acid inducible gene - I (RIG-I)+TLR7	3p-dsRNA	-
cytosolic DNA receptro+RIG-I	dA:dT	-

## Materials and Methods

### DNA-, RNA-oligonucleotides and TLR ligands

CpG ODN were purchased from Metabion (Martinsried, Germany) (capital letters = phosphodiester linkage, small letters = phosphorothioate linkage): CpG 2006 (5′-tcgtcgttttgtcgttttgtcgtt); CpG M362 (5′-tcgtcgtcgttcgaacgacgttgat); neg contr. – C20-DNA (5′-cccccccccccccccccccc). RNA ODN were purchased from Biomers (Ulm, Germany) 9.2s (5′-AGCUUAACCUGUCCUUCAA) [4]; eGFP siRNA [28] (underlined letters = 2′-O ribose methylation): ss-eGFP-sense (no meth. 5′-GACGUAAACGGCCACAAGUUC); ss-eGFP-s3methylated (3x meth 5′-GACGUAAACGGCCACAAGUUC); ss-eGFP-as (5′-ACUUGUGGCCGUUUACGUCGC), neg. contr. – A20-RNA (5′-AAAAAAAAAAAAAAAAAAAA) and dAdT from Sigma-Aldrich (St. Louis, MO). For generation of DNA-template-dependent *in vitro*-transcribed RNA (3p-dsRNA), the T7-promoter region 5′-CAGTAATAGGACTCACTATAG-3′ was hybridized with the promoter+template strand (5′- TTGTAATACGACTCACTATAGGGACGCTGACCCAGAAGATCTACTAGAAATAGTAGATCTTCTGGGTCAGCGTCCC) and directly used as a template for *in vitro* transcription reactions. R848 was obtained from Alexis (Lörrach, Germany), *E.coli* LPS from Sigma-Aldrich (St. Louis, MO).

### Ethics Statement

The PBMC and whole blood studies were conducted according to the principles of the Declaration of Helsinki and were approved by the responsible ethics committee (Ethics committee of the Medical Faculty, University of Bonn) before start. Human whole blood was drawn from healthy volunteers after obtaining written informed consent. Buffy Coats were obtained from the Institute of Transfusion Medicine at the University of Bonn. Blood donors gave their written informed consent that buffy coats were used for research purpose.

### Preparation, isolation and culture of cells

Human whole blood was drawn from healthy volunteers using standard butterflies and blood drawing system from Sarstedt (Nümbrecht, Germany) with EDTA or Heparin-containing monovettes or 50 ml syringes from Braun (Melsungen, Germany) prefilled with 1 mg Hirudin (Refludan® from Pharmion/Celgene, Munich, Germany). 200 µl of whole blood were cultured in 96-well plates (TPP, Trasadingen, Swiss). Freshly prepared buffy coats from human donors were obtained from the Institute for Experimental Hematology and Transfusion Medicine, University Hospital of Bonn, Bonn, Germany with the donors' written informed consent. PBMCs were prepared by density gradient centrifugation using Ficoll separating solution (Biochrom, Cambridge, U.K.) and lysis of RBCs using BD Pharm Lyse (BD Pharmingen). Cell viability exceeded 95% as examined by trypan blue staining. 4×10^5^ PBMCs were cultured in 200 µl RPMI 1640 (Biochrom, Berlin, Germany) 10% (v/v) FCS (Invitrogen, Karlsruhe, Germany), 1 mM L-Glutamine, 100 U/ml penicillin and 100 µg/ml streptomycin (Sigma-Aldrich, St. Louis, MO) in 96-well plates.

### Stimulation of cells

Cells were stimulated in duplicate with TLR ligands and oligonucleotides in supernatant in concentrations as indicated. Alternatively oligonucleotides were added to the cells together with appropriate delivery agents known to reach the cellular compartment of the targeted receptor (endosome: pL-Arginine/Protamine/DOTAP/JetPEI versus cytosol: Lipofectamine2000/JetPEI). In brief, each 200 ng RNA/DNA complex was incubated with: 0.5 µl Lipofectamine2000 (Invitrogen, Karlsruhe, Germany) for 15 min/RT in 50 µl OptiMEM (Invitrogen, Karlsruhe, Germany); 360 ng pL-Arginine (pArg) (Sigma-Aldrich, St. Louis, MO) for 10 min/RT in 15 µl PBS; 2 µl DOTAP (Carl Roth, Karlsruhe, Germany) for 10 min/RT in 50 µl OptiMEM, 360 ng Protamine (Sigma-Aldrich, St. Louis, MO) for 1 min/RT in 15 µl PBS, 0.016 µl invivoJetPEI (Polyplus, Saint Quentin Yvelines, France) for 20 min/RT in 50 µl 5% Glucose and then added to the cells.

### ELISA

After indicated time points cytokines were measured in cell culture supernatants by the IFN alpha module set (ebiosience), BD OptEIA TNF and BD OptEIA IL-6 ELISA kits (BD Biosciences, Heidelberg, Germany) according to manufacturer's recommendations.

### Oligonucleotide release assay

CpG 2006, 9.2 s and 3p-dsRNA were incubated with corresponding delivery agent as described above. After 20 min, Heparin (20 IE/ml) or Hirudin (20 µg/ml) were added in the same concentration as used for stimulation (see above) for 40 min, followed by DNase I (Fermentas, St. Leon Rot, Germany) or RNase A (Fermentas, St. Leon Rot, Germany) digestion at 37°C (CpG-ODN/pArg 4 U DNase I 4 h; CpG-ODN/DOTAP 3 U DNase I 4 h; 9.2 s-RNA/DOTAP 250 ng RNase A 10 min; 3p-dsRNA/Lipofectamine 500 ng RNase A 90 min). RNase A was inactivated by adding 280 U RiboLock (Fermentas, St. Leon Rot, Germany) for 15 min and DNase I by heating to 65°C for 10 min. After addition of Heparin to displace all nucleic acids from delivery agent, the mixture was loaded on a native polyacrylamide gel (15%). DNA/RNA was stained with SYBR®-Green II RNA Gel Stain (Invitrogen, Karlsruhe, Germany).

### Statistical analysis

Statistical analysis was performed using student's t-test for dependent samples or one-sample t-test in case of relative comparison. In case of multiple comparison Bonferroni correction was applied or alternatively One-way analysis of variance (ANOVA) followed by corrected post-hoc test was used (SPSS software). P-values of less than 0.05 were considered statistically significant.

## Results

### The whole blood assay (WBA) with Heparin or Hirudin and the PBMC assay show comparable cytokine induction by LPS and R848

**Figure 1:**
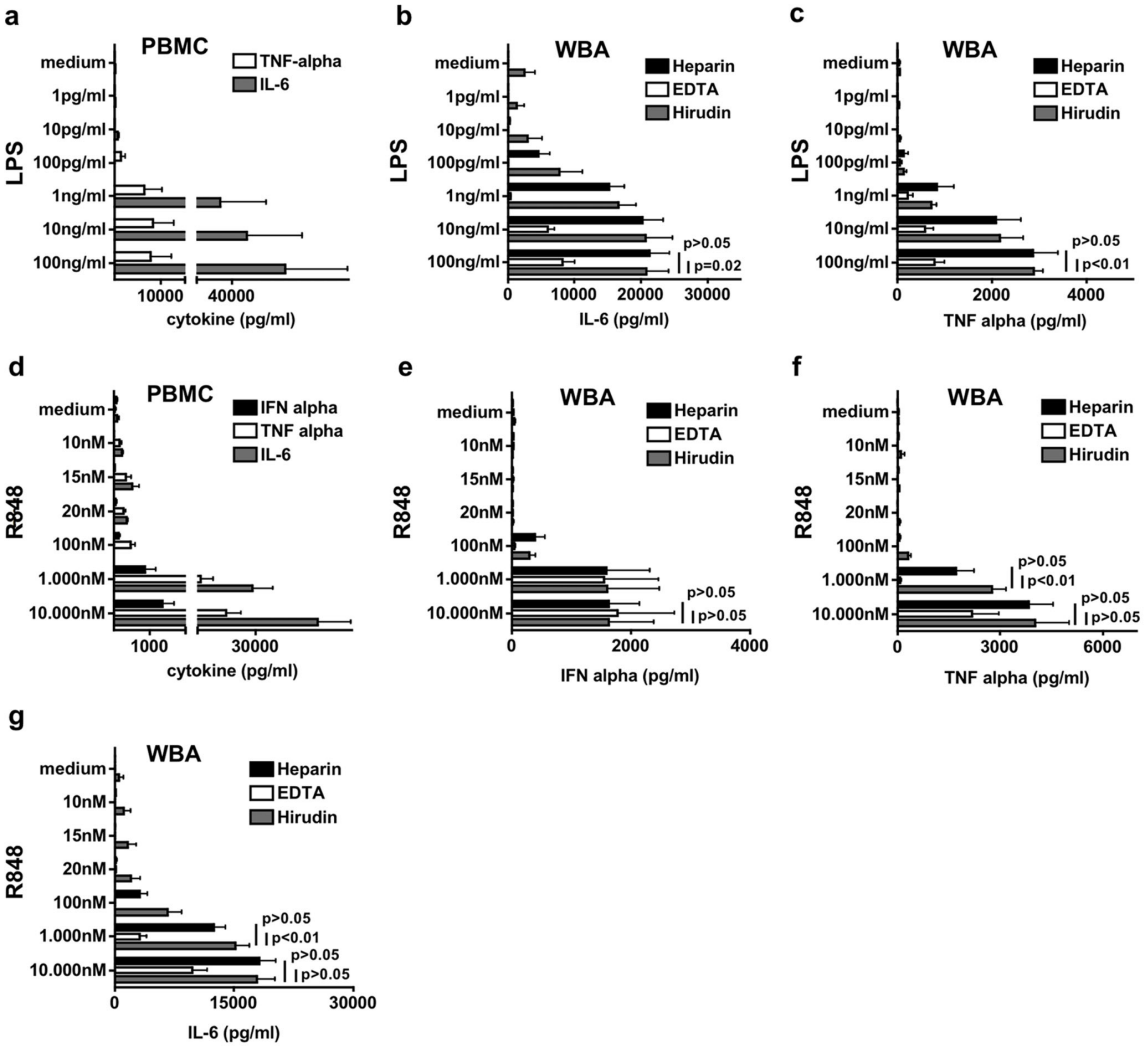
Comparison of cytokines induced by LPS and R848 in PBMC and in a human WBA. a – PBMC were incubated in 96 well with increasing concentrations of LPS. After 48 h supernatant was analyzed for TNF-α and IL-6 using ELISA. Results of eight donors are shown as mean +/− SD. b–c – Whole blood anticoagulated either with Heparin, EDTA or Hirudin was incubated in 96 wells with increasing concentrations of LPS and after 48 h supernatant was checked for IL-6 (b), TNF-α (c). Mean of six donors is shown +/− SD. d – Done as described in (a) but R848 was used for stimulation and supernatant was analyzed for IFN-α, TNF-α and IL-6. Mean of eight donors (three for IFN-α) is shown +/− SD. e–g – Done as described for (b) but R848 was used for stimulation and IFN-α (e), TNF-α (f) and IL-6 (g) were measured in supernatant. Results of four (IFN-α) and six donors are shown as mean +/− SD.

As the first step towards a whole blood assay (WBA) that identifies cytokine release induced by therapeutic oligonucleotides, we started with established non-RNA/DNA ligands targeting nucleic acid recognizing- or endotoxin recognizing TLRs. We used the small molecule resiquimod (R848) acting on the RNA-detecting receptors TLR7 and 8 as well as LPS, known to activate TLR4. We compared cytokine induction by R848 and LPS in PBMC with whole blood anticoagulated with Heparin, EDTA or Hirudin (Fig. 1). Induction of typical cytokines in response to LPS (TNF-α, IL-6; Fig. 1a–c) and R848 (IFN-α, TNF-α, IL-6; Fig. 1d–g) were seen in both assays. Although induced cytokine concentrations were in general higher in PBMC, the threshold concentration of agents LPS or R848 leading to detectible induction of cytokines was comparable in both assays, as long as Heparin or Hirudin were used as anticoagulant. By contrast, EDTA anticoagulated blood showed a reduced response to the stimuli. Thus, the WBA assay with Heparin or Hirudin as anticoagulant yields the same qualitative results for cytokine induction in response to non-oligonucleotide TLR4/7/8 ligands as the established but more time consuming PBMC assay.

### The *in vitro* whole blood assay simulates IFN-α induction by CpG-ODN *in vivo* more accurately than the PBMC assay

**Figure 2:**
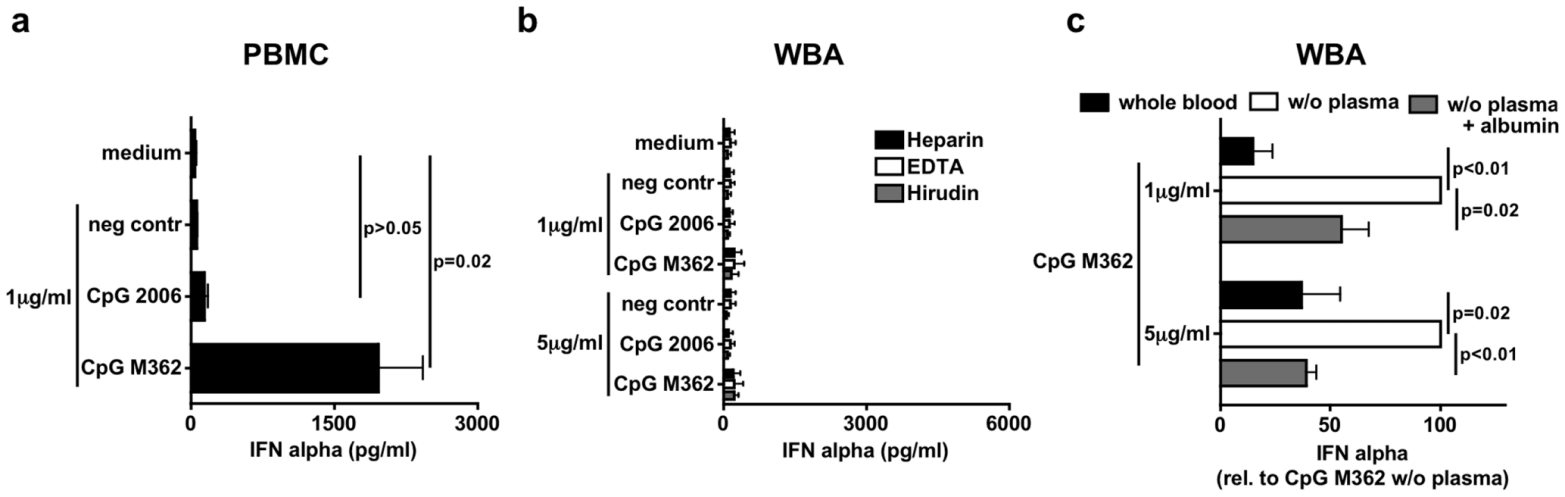
The WBA simulates IFN-α induction by CpG-ODN in vivo more accurately than the PBMC assay. a – PBMC were stimulated with B-type (CpG 2006) and C-type (M362) CpG-oligonucleotides as indicated. C20-DNA served as a negative control (neg contr). After 48 h induction of IFN-α was analyzed using ELISA. b – Done as described for (a) but whole blood anticoagulated with Heparin, EDTA or Hirudin was used. c – Complete whole blood or whole blood in which plasma was replaced by medium or medium+albumin was anticoagulated with Hirudin and stimulated with B-type (CpG 2006) and C-type (CpG M362) CpG-oligonucleotides or C20-DNA (neg contr) as indicated. After 48 h supernatant was analyzed for IFN-α concentration. Graph shows percentage of induced IFN-α compared to CpG M362 (induced IFN-α concentrations: 1 µg/ml CpG M362: 1697 pg/ml; 5 µg/ml CpG M362: 3627 pg/ml). Results of at least six donors are shown as mean +/− SD in (a–c).

Next, we analyzed immune stimulation by oligonucleotides, starting with CpG-oligodesoxynucleotides (CpG-ODN), an established ligand for TLR9 already in clinical development. Again we compared PBMC (Fig. 2a) with the WBA (Fig. 2b) using two classes of CpG-ODN that have been already tested in clinical trials: B-type CpG-ODN (CpG 2006, also known as CpG 7909 and ProMune®) that activates B-cells and C-type CpG-ODN (CpG M362, also known as CpG 10101 and Actilon®) that additionally induces IFN-α in PDC *in vitro*. In contrast to R848 and LPS, CpG-ODN showed a significant difference in both assays: In PBMC C-type CpG-ODN but not B-type ODN induced IFN-α (Fig. 2a). Conversely, in WBA there was no measurable IFN-α induction by both classes of CpG-ODN irrespective of the anticoagulant used (Fig. 2b). The results obtained with the WBA reflect the reported *in vivo* results for these CpG-ODN in clinical trials: Intravenous application of CpG 2006 did not induce IFN-α up to the highest dose tested [29] and although a subcutaneous application of CpG M362 led to measurable drug levels in the plasma of healthy volunteers, almost no IFN-α was detectable [30]. It is known that CpG-DNA binds to a high degree to plasma proteins, which could be speculated as reason for inactivity of CpG-DNA in the blood [2]. To test this hypothesis we replaced the plasma in the WBA by medium or by medium supplemented with physiological concentrations of the main plasma protein albumin before stimulation with CpG-ODN (Fig. 2c). When plasma was exchanged with medium CpG-ODN C-type (CpG M362) but not B-type (CpG 2006, data not shown) was able to induce IFN-α (Fig. 2c) as seen in and published for PBMC (Fig. 2a) [6]. This was again strongly reduced after addition of albumin. These findings suggest that indeed plasma proteins like albumin are responsible for inhibition of a CpG-ODN mediated stimulation of immune cells. The data indicate that the cytokine induction seen in the WBA assay mirrors the intravenous *in vivo* application of CpG-ODN in humans more precisely than the cytokine induction obtained in the PBMC assay.

### Amplification of the nucleic acid induced IFN-α response by transfection reagents tolerates Hirudin- but not Heparin or EDTA as anticoagulant in the whole blood assay

**Figure 3:**
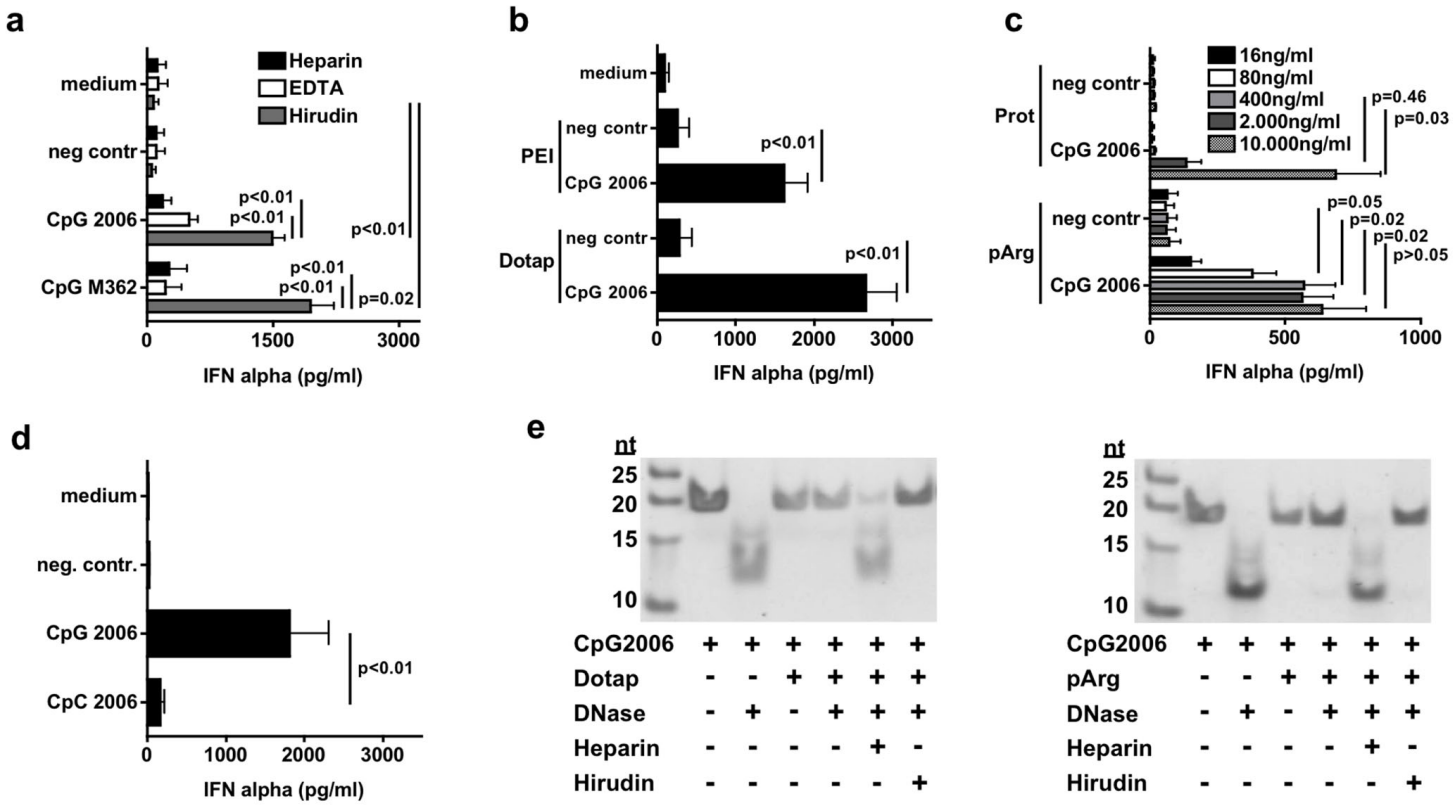
Amplification by transfection tolerates anticoagulation with Hirudin but not Heparin or EDTA in the WBA. a – Whole blood was anticoagulated with Heparin, EDTA or Hirudin and stimulated with 0.8 µg/ml B-type (2006) or C-type (M362) CpG-oligonucleotide or C20-DNA (negative control) in complex with DOTAP. After 48 h supernatant was analyzed for IFN-α induction by ELISA. Results of at least six donors are shown as mean +/− SD. b – Done as described for (a) but additional polyethylenimine (PEI) was used as delivery agent. Results of at least four donors are shown as mean +/− SD. c – Done as described for (a) but Protamine (Prot) and poly-L-Arginine (pArg) were used as delivery agents in complex with increasing concentrations of DNA as indicated. Results of at least eight donors are shown as mean +/− SD. d – Whole blood was anticoagulated with Hirudin and stimulated with 0.8 µg/ml CpG 2006 or CpC 2006 (CpG replaced by CpC sequences), both delivered with DOTAP. C20-DNA served as negative control. After 48 h supernatant was analyzed for IFN-α by ELISA. Results of eight donors are shown as mean +/− SD. e – CpG 2006 was incubated with DOTAP or pArg with or without addition of Heparin or Hirudin and digested with DNase before gel electrophoresis.

Enforced delivery to the endosomally located TLR9 by transfection reagents is known to enhance the activity of CpG-ODN [31]–[33]. Therefore we tested whether complexation with transfection reagents overcomes the inhibition of plasma and enables CpG-ODN to induce IFN-α in the WBA. If Hirudin was used as the anti-coagulant, delivery with DOTAP turned the B type and C type CpG-ODN into robust inducers of IFN-α (Fig. 3a). By contrast, EDTA or Heparin nearly completely abolished IFN-α induction by DOTAP+CpG-ODN (Fig. 3a). To show that this is a general effect and not only mediated by DOTAP we tested polyethylenimine (PEI) (Fig. 3b) as well as the polypeptides poly-L-Arginine (pArg) and Protamine (Prot) (Fig. 3c) as delivery agents. Despite differences in efficacy, all tested agents were able to mediate IFN-α induction by CpG-ODN. To demonstrate that formulated CpG-ODN still functions via TLR9 in the WBA we replaced the CG motif by CC, which led to a loss of IFN-α induction in line with TLR9 dependency even in combination with a delivery agent (Fig. 3d). To uncover the reason why anticoagulation with Hirudin but not with Heparin allows induction of IFN-α, we digested CpG-ODN with DNase after particle formation with the delivery agent. Dotap or pArg were able to protect CpG-ODN from digestion by DNase either in absence of anticoagulant or if Hirudin was added, but not in the presence of Heparin (Fig. 3e). This indicates that Heparin inhibits immune activation by displacing the delivery agent from the DNA, which becomes accessible for DNases. Taken together, usage of Hirudin as an anticoagulant in the WBA warrants the integrity of particles formed by delivery agents with CpG-ODN and maintains potent IFN-α induction independently of the class of CpG-ODN.

### The Hirudin anticoagulated WBA exhibits high reliability of immune activation pattern upon repeated testing of the same donor

**Figure 4:**
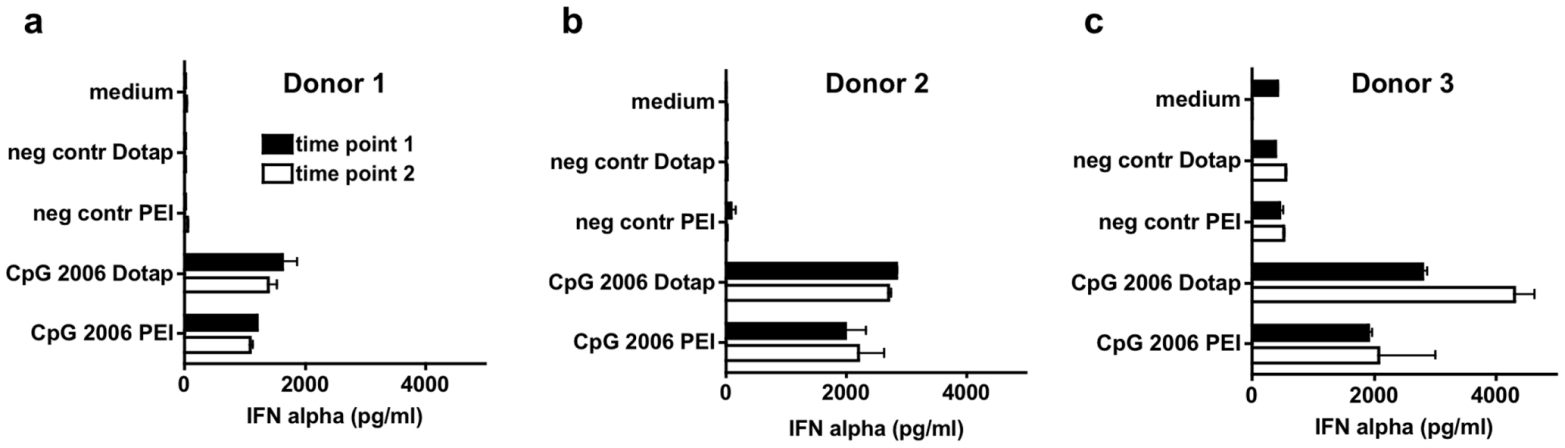
The Hirudin anticoagulated WBA exhibits high reliability upon repeated testing of the same donor. Whole blood of three different donors (a–c) anticoagulated with Hirudin was stimulated twice with one week inbetween with 0.8 µg/ml CpG 2006 delivered with polyethylenimine (PEI) or DOTAP. C20-DNA served as negative control. After 48 h supernatant was analyzed for IFN-α by ELISA. Mean of duplicates +/− SD is shown for each timepoint.

To test the repeatability of IFN-α induction with complexed CpG-ODN in the WBA we compared the variation between different donors and within one donor over time. Three different donors showed clear IFN-α induction upon stimulation with CpG-ODN (Fig. 4a–c). However, the IFN-α concentrations achieved varied between donors tested, arguing for individual differences in the response to TLR9 activation. After approximately one week the WBA was repeated in the same donors not reporting or exhibiting obvious changes in immune and health status (e.g. respiratory infections). The individual blood donor-specific immune activation pattern of IFN-α could be repeated, demonstrating reproducibility of the assay.

### Hirudin-whole blood assay – application as a fast and easy screening method to determine immunostimulatory side effects of siRNA therapeutics

**Figure 5:**
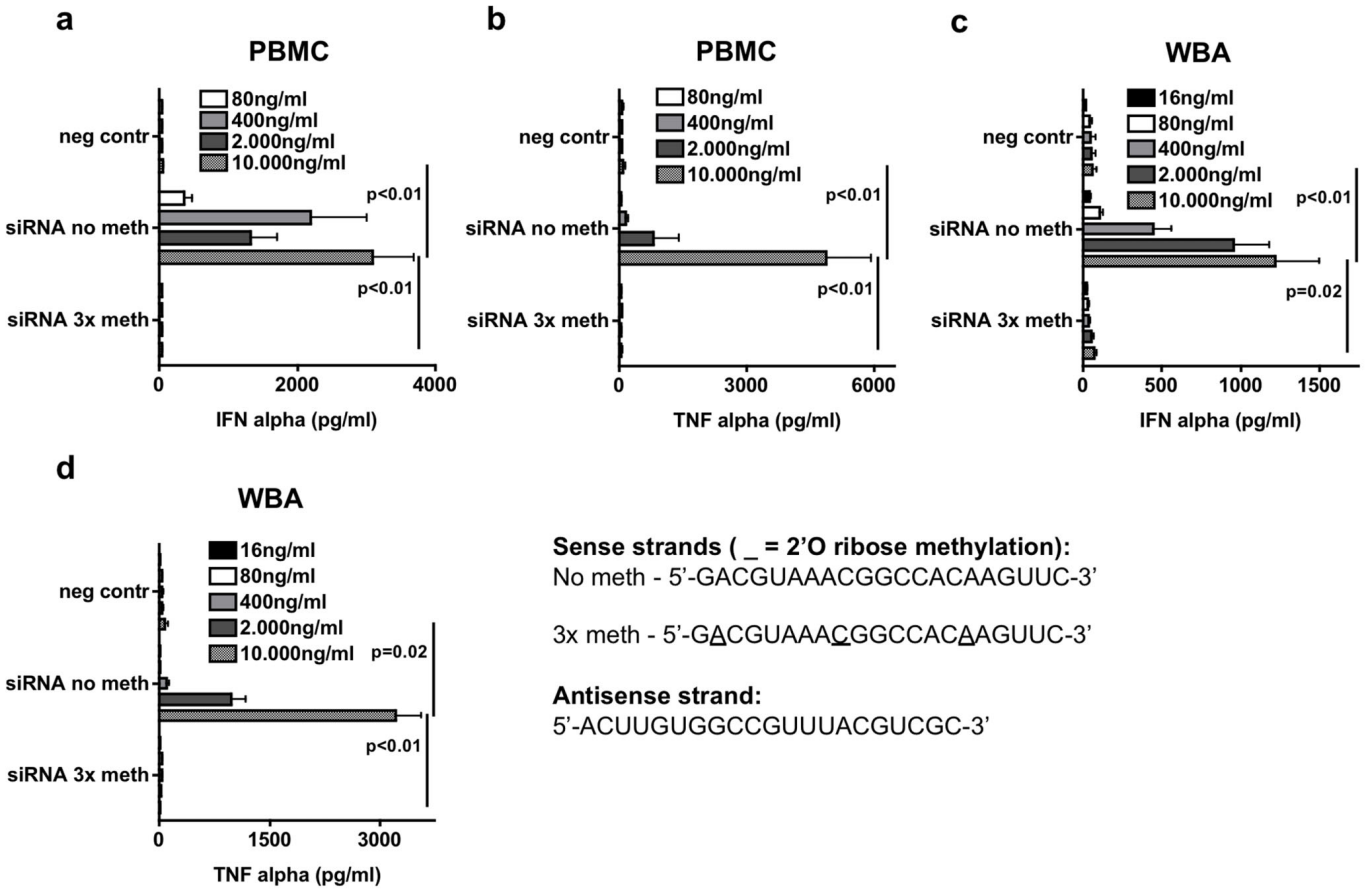
Hirudin-WBA – application as fast screening method to determine immunostimulatory side effects of siRNA therapeutics. a–b – PBMC were stimulated with siRNA without (no meth) or with three 2′-O ribose methylated nucleotides (3x meth) delivered with p-L-Arginine (pArg) in increasing concentrations as indicated. After 48 h concentrations of IFN-α (a) and TNF-α (b) in supernatant was analyzed by ELISA. c–d – Done as described for (a) and (b) but whole blood assay anticoagulated with Hirudin was used. Results of eight donors are shown as mean +/− SD in (a–d). For sequence details of siRNA used see lower right paragraph.

siRNA is a promising class of therapeutic oligonucleotides [1]. If not given strictly locally, every siRNA needs to be checked for systemic immunactivation in the blood as an unwanted non-intended effect [4], [11]. Different strategies have been evaluated to circumvent the potential recognition of siRNA by TLR7, e.g. methylation of nucleotides [28]. We wanted to test whether the Hirudin-WBA is able to reproduce the results of TLR7 activation by siRNA seen in PBMC. We took advantage of a published eGFP-siRNA that shows TLR7 induction dependent on integrated 2′-O methylated nucleotides [28]. As described, methylation of 3 nucleotides (3x meth) inhibited the ability to induce IFN-α and TNF-α in PBMC compared to unmethylated siRNA (no meth) (Fig. 5a,b). The same was true for the Hirudin-WBA (Fig. 5c+d). As seen for LPS and R848 (Fig. 1), the induced amounts of cytokines were higher in PBMC than in the Hirudin-WBA but the threshold was at comparable concentrations. Taken together, the Hirudin-WBA is a fast and easy to perform screening assay to test siRNA on its immunostimulatory potential.

### RNA ligands for TLR7 and RIG-I induce IFN-α in Hirudin-whole blood assay when added with a delivery agent

**Figure 6:**
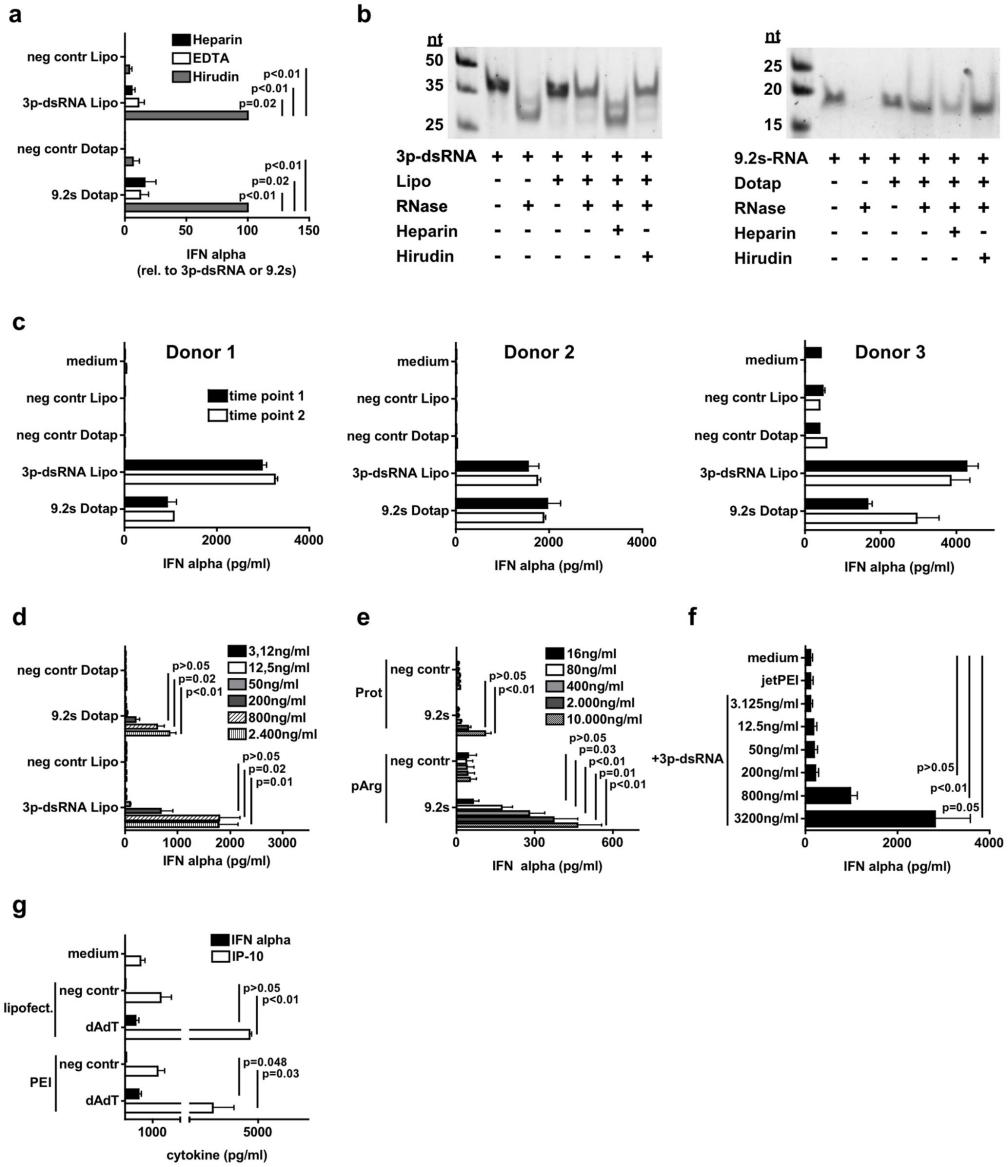
RNA ligands for TLR7 and RIG-I induce IFN-α in Hirudin-whole blood assay. a – Whole blood was anticoagulated with Heparin, EDTA or Hirudin and stimulated with 0.8 µg/ml 3p-dsRNA, 9.2s-RNA or A20-RNA (negative control) delivered with Lipofectamine2000 or DOTAP. After 48 h supernatant was analyzed for IFN-α induction by ELISA. Graph shows percentage of induced IFN-α compared to 3p-dsRNA and 9.2s-RNA respectively (induced IFN-α concentrations: 3p-dsRNA: 2641 pg/ml; 9.2s-RNA: 1438 pg/ml). Results of four donors are shown as mean +/− SD. b – 3p-dsRNA was incubated with Lipofectamine2000 and 9.2s-RNA with DOTAP with or without addition of Heparin or Hirudin. Complexes were digested by RNase and subsequently analyzed by gel electrophoresis. c – Whole blood of three different donors anticoagulated with Hirudin was stimulated at two different time points with 0.8 µg/ml 3p-dsRNA, 9.2s-RNA or A20-RNA (negative control). After 48 h supernatant was analyzed for IFN-α using ELISA. Mean of duplicates +/− SD are shown for each timepoint. d – Whole blood was anticoagulated with Hirudin and stimulated with increasing concentrations of 3p-dsRNA and 9.2s-RNA. Results of eight donors are shown as mean +/− SD. e – Done as described for (d) but Protamine (Prot) and poly-L-Arginine (pArg) were used as delivery agents for increasing concentrations of 9.2s-RNA. Results of eight donors are shown as mean +/− SD. f – Done as described for (d) but polyethylenimine (PEI) was used to deliver increasing concentrations of 3p-dsRNA. Results of six donors are shown as mean +/− SD. g – Done as described for (d) but 0.8 µg/ml of dAdT was used to stimulate the cells. Results show mean of IFN-α and IP-10 induction of six donors +/− SD.

After establishing a WBA analyzing cytokine release by established ligands and oligonucleotides, we wanted to test RNA-oligonucleotides not yet approved for use in humans. We used a RNA-oligonucleotide (9.2sRNA) targeting TLR7/8 located in the endosome and 3p-dsRNA known to activate the cytosolic receptor RIG-I+the endosomal TLR7, both interesting candidates for the development of immunostimulatory, type I IFN inducing drugs [3]. In complex with appropriate delivery agents (targeting the endosome (DOTAP, pArg, Protamine) versus the cytosol (Lipofectamine)) 9.2sRNA as well as 3p-dsRNA were able to induce IFN-α in the Hirudin WBA in comparable amounts as CpG-ODN (Fig. 6a). Again, Heparin inhibited IFN-α induction by release of RNA from particle formed with delivery agent whereas Hirudin left the complex intact (Fig. 6a+b). As observed for CpG-ODN (Fig. 4) we detected IFN-α induction in all donors tested with relevant inter-individual differences but low variations within the same donor upon repeated testing (Fig. 6c) again confirming the reproducibility of the assay also for RNA based immune stimuli. Since 9.2sRNA and 3p-dsRNA have not been used in humans, careful determination of a safe starting dose, an anticipated active dose and definition of the dose-response relationship is required before going into clinical testing. We performed titrations to determine the minimal effective concentration and the response to higher concentrations in terms of IFN-α induction in the Hirudin-WBA. 3p-dsRNA and 9.2s-RNA showed a linear concentration dependent induction of IFN-α with a minimal concentration of 200–800 ng/ml for 3p-dsRNA and 9.2s-RNA leading to IFN-α induction (Fig. 6d). We compared different delivery strategies for 9.2s-RNA (Fig. 6d+e) and 3p-dsRNA (Fig. 6d+f) demonstrating that the minimal concentration inducing IFN-α is strongly dependent on the delivery agent (9.2s: DOTAP 200–800 ng/ml; pArg 80–400 ng/ml; Protamine 2000–10000 ng/ml; 3p-dsRNA: Lipo and PEI 200–800 ng/ml). Since 3p-dsRNA can activate both cytosolic RIG-I and endosomal TLR7, we wanted to prove that cytosolic activation can be monitored by the WBA using a pure cytosolic ligand. We used the DNA molecule dAdT that is known to activate a cytosolic DNA receptor but not TLR9, and probably also RIG-I in human cells [34]–[36]. Still, IFN-α and IP-10 was induced (Fig. 6g), indicating that cytosolic activation can be achieved and monitored in the WBA. Taken together, 3p-dsRNA and 9.2sRNA are able to induce IFN-α in a robust and linear concentration dependent fashion in the Hirudin-WBA with a minimal biological effect level dependent on the used delivery agent.

## Discussion

In preclinical testing of a new investigational medicinal product (IMP) it is crucial to find the most meaningful assays to estimate a safe starting dose and safe dose escalation steps. Due to the Tegenero desaster [13] it became aware that the life-threatening side effects of excessive cytokine release by an immune activating drug is difficult to predict since the immune system is highly species specific. Therefore, apart from *in vivo* tests in animals, *in vitro* assays involving human specimen are needed to characterize and predict the cytokine release induced by new drugs in the human system as precisely as possible. In addition to the more established PBMC assay a human whole blood assay has been suggested for that purpose [37] and different classes of drugs have been already tested successfully [14]–[17], [21], [22], [37]. While there are a few reports on analysis of TLR ligands in the WBA, studies including oligonucleotides with or without a delivery agent and focussing on cytokine release are rare or lacking [18], [19], [23], [38]. Since most of the therapeutic oligonucleotides either function via immune activation and cytokine release (e.g. TLR-ligands, RLR-ligands), or immune activation is considered a possible unwanted side effect (e.g. siRNA via TLR7), it is of special interest to have a reliable human *in vitro* test to investigate cytokine release by this class of drugs. We observed that the WBA possess the same threshold for cytokine induction by LPS and R848, TLR4 and TLR7/8 ligands respectively, as the more time consuming PBMC assay. The WBA, but not the PBMC assay, mirrored the *in vivo* situation seen with CpG-DNA (TLR9 ligand): According to previous studies, after i.v. application of the B-type CpG 2006 no IFN-α induction is detectable *in vivo* [29] and the C-type CpG M362 – even if measurable in the plasma – induces only marginal plasma levels of IFN-α *in vivo* [30]. The low IFN-α induction *in vivo* was mirrored in our WBA experiments. By contrast, IFN-α was substantially induced in PBMC by C-type CpG. Substitution of plasma by medium in the WBA led to substantial CpG-DNA mediated cytokine induction. Addition of albumin to the medium abolished cyokine induction again. It is known that CpG-DNA is bound by plasma proteins [2]. Segregation of naked oligonucleotides by binding to plasma albumin could explain why CpG-DNA does not induce cytokines *in vivo* after i.v. application. Altogether, the data suggest that the WBA reflects broad aspects of the *in vivo* cytokine release by oligonucleotides and small molecule TLR stimuli even more consistently than the more established PBMC assay. When complexed to a delivery agent, the cytokine release by CpG-DNA was enhanced in the WBA. All types of CpG-DNA induced substantial amounts of IFN-α, the main therapeutically active cytokine. This parallels reports showing an enhanced therapeutic effect by CpG-DNA after enforced delivery by transfection reagents [31]–[33] and indicates how the efficacy of CpG-DNA can be increased [2]. After establishing the WBA we also tested immune activating RNAs, which have not been tested in humans so far. We found that a TLR7/8 ligand (9.2s) and a combined RIG-I+TLR7-ligand (3p-dsRNA) were able to induce substantial amounts of IFN-α, arguing for a potential efficacy in viral diseases and cancer. A titration showed a linear concentration-dependent effect dependent on the delivery agent. Using dAdT as a ligand for a cytosolic DNA receptor capable of inducing type I IFN probably also via RIG-I [36] but not via TLR9 we could show that cytosolic activation is indeed achieved and can be monitored in the WBA. In combination with *in vivo* results, these data can be used to calculate a safe starting dose or to predict the efficacy of the compound when entering the clinical development. A known side effect of siRNA used to knock down a pathophysiology-related protein is to induce cytokines, especially via TLR7 and 8 [11]. Therefore, siRNAs have to be tested for immune activation before treatment of humans. We compared a non-methylated with a methylated siRNA that has been characterized for TLR7/8 activation in PBMC [28]. We found that methylation avoids cytokine release by siRNA in PBMC as well as in WBA. Regardless whether CpG-DNA or RNA was used, the amount of IFN-α induction was dependent on the individual blood donor but did not change within one individual over time, indicating a good reliability of the WBA. The anticoagulant used in a WBA for therapeutic oligonucleotides is critical. Heparin and Hirudin, but not EDTA, allowed cytokine induction with LPS, R848 and CpG-ODN not combined with a delivery agent. Moreover, only Hirudin did not interfere with cytokine induction by nucleic acid stimuli formulated in complex with a delivery agent, whereas negatively charged Heparin displaced the delivery agent from nucleic acids. There are limitations of the WBA that needs to be considered. It cannot replace *in vivo* tests since it just mimics the consequences on the compartment blood and does not reflect a more complex *in vivo* situation with a drug that induces cytokines in different compartments. Additionally, the WBA does not simulate an *in vivo* course of the plasma concentration curve. There is no gradual increase, peak concentration and gradual decrease of the drug, possibly leading to different results in the WBA and in vivo. Whether the WBA can be additionally used to predict the anaphylactic reaction and complement activation by oligonucleotides is not covered in this work and thus needs to be investigated in future studies. If these limitations are taken into account, our results demonstrate that the WBA is a fast and suitable assay to predict the induction of cytokine release in the human blood by therapeutic oligonucleotides. Since it is faster and cheaper and, according to our data, more representative for i.v. applied therapeutic oligonucleotides in vivo than the PBMC assay, we suggest to include the Hirudin WBA into the preclinical program of therapeutic oligonucleotides.
